# Synergistic Effects of Solar Exposure and Skin Sensitivity on Skin Aging: A Clinical Study Using Multimodal Noninvasive Measurements

**DOI:** 10.1111/srt.70389

**Published:** 2026-10-01

**Authors:** Du Shan, Lin Guihua, Zhong Xinqing, Shi Jiajia, YunHa Lee, Xiong Zhi

**Affiliations:** ^1^ AMOREPACIFIC (SHANGHAI) D&I CENTER CO., LTD Shanghai PR China

**Keywords:** interaction effect, noninvasive measurement, photoaging, sensitive skin, skin aging, solar exposure

## Abstract

**Background:**

Solar exposure is the primary extrinsic factor contributing to skin aging. Sensitive skin, characterized by impaired barrier function and heightened reactivity, may be more vulnerable to solar‐induced damage. However, the potential interaction between skin sensitivity and solar exposure in accelerating skin aging has not been systematically quantified using objective clinical measurements.

**Objectives:**

To investigate the interactive effects of skin sensitivity and solar exposure on multiple aging‐related skin parameters using multimodal noninvasive measurement techniques.

**Methods:**

This single‐center observational study enrolled 124 healthy Chinese female volunteers aged 25–34 years. Participants were categorized according to skin sensitivity (sensitive vs. nonsensitive) and occupational solar exposure (indoor vs. outdoor), forming four groups (*n* = 31 per group): indoor sensitive (IS), outdoor sensitive (OS), indoor nonsensitive (INS), and outdoor nonsensitive (ONS). Skin barrier function, surface morphology, elasticity, dermal structure, and sebum secretion were evaluated using a series of noninvasive instruments. Two‐way analysis of variance (ANOVA) was used to assess the main effects and interaction effects of skin sensitivity and solar exposure.

**Results:**

Significant interaction effects between skin sensitivity and solar exposure were observed for seven aging‐related parameters, including transepidermal water loss (TEWL), stratum corneum thickness (SCT), second harmonic generation (SHG), autofluorescence (AF), skin roughness, nasolabial fold length, and sebum secretion (*p* < 0.05). The outdoor sensitive group exhibited the most pronounced aging phenotype, characterized by increased TEWL, reduced stratum corneum thickness, decreased SHG and AF signals, greater skin roughness, and longer nasolabial folds.

**Conclusion:**

Skin sensitivity and solar exposure exhibit a significant synergistic effect in the skin aging process. Individuals with sensitive skin under chronic solar exposure demonstrate more severe barrier dysfunction, dermal matrix degradation, and wrinkle formation. These findings suggest that sensitive skin represents a high‐risk phenotype for photoaging and highlight the importance of combined barrier repair and effective photoprotection strategies for this population.

## 1 Introduction

Skin aging is a complex biological process driven by both intrinsic genetic factors and extrinsic environmental influences [1]. Among environmental factors, chronic solar radiation exposure is considered the most important contributor to extrinsic aging, commonly referred to as photoaging. Clinically, photoaged skin is characterized by wrinkles, surface roughness, pigmentation irregularities, decreased elasticity, and impairment of epidermal barrier function [2].

Sensitive skin is a common condition characterized by unpleasant sensory symptoms such as burning, stinging, itching, and tightness in response to stimuli that are normally well tolerated. Epidemiological studies have consistently reported a high prevalence of sensitive skin worldwide [3, 4]. Current evidence suggests that sensitive skin is associated with several physiological alterations, including impaired skin barrier function [5, 6, 7], neurosensory dysregulation [8, 9, 10], and increased vascular reactivity [11, 12].

In particular, barrier dysfunction is considered a key pathological feature of sensitive skin [13].Structural abnormalities in the stratum corneum may reduce the skin's defensive capacity against environmental stressors, allowing exogenous irritants and physical stimuli, including solar radiation, to penetrate the epidermis more readily. This increased permeability may induce stronger oxidative stress responses and inflammatory signaling pathways, which may clinically present as erythema, irritation, and discomfort [14].

Emerging evidence indicates that the pathophysiological mechanisms of sensitive skin and skin aging are intimately intertwined, with shared hallmarks including barrier impairment, chronic low‑grade inflammation, and extracellular matrix degradation [15]. Indeed, the compromised barrier function in sensitive skin not only facilitates the penetration of environmental irritants but also reduces its intrinsic anti‑aging capacity, thereby accelerating the aging process [16, 17].Furthermore, sensitive skin may undergo accelerated collagen degradation and impaired cellular function as a consequence of long‐term inflammatory responses, both of which are closely associated with skin aging [18].

Given these characteristics, individuals with sensitive skin may exhibit increased vulnerability to environmental damage, potentially hastening the appearance of visible aging signs. However, clinical evidence directly linking skin sensitivity to accelerated photoaging remains scarce.

In this context, several recent studies have begun to explore the potential link between sensitive skin and premature aging.  A large‑scale study involving 810 Chinese participants demonstrated that environmentally triggered sensitive skin was significantly associated with pigment spots on the cheeks, whereas intrinsically triggered sensitive skin was correlated with perioral wrinkles, providing the first objective evidence linking specific sensitive‑skin subtypes to distinct skin‑aging phenotypes [19].

 Our previous research demonstrated that individuals with sensitive skin aged 25–29 years exhibited earlier aging characteristics compared with nonsensitive individuals, including larger facial pores, more pronounced nasolabial folds, reduced skin transparency, and higher AI‐predicted skin age. Interestingly, these differences were more pronounced in younger age groups [20].

Furthermore, proteomic analysis [21] revealed substantial molecular differences between sensitive and nonsensitive skin, including reduced barrier‐related proteins, increased oxidative stress markers, elevated DNA damage signals, dysregulated neuro‐immune signaling, and altered lipid metabolism. Mechanistically, recent studies have identified CCN1 (Cellular Communication Network Factor 1) as a critical molecular mediator bridging sensitive skin and skin aging. CCN1 is markedly upregulated by ultraviolet radiation and oxidative stress, and its sustained elevation can simultaneously impair barrier function, amplify inflammation, drive collagen degradation, and promote fibroblast senescence—establishing a self‐reinforcing cycle in which barrier defects accelerate aging, while aged skin becomes more fragile and reactive [15] These molecular changes suggest that sensitive skin exists in a state of chronic physiological stress, which may predispose it to accelerated aging processes when exposed to environmental stressors.

Despite these findings, the potential synergistic interaction between solar exposure and skin sensitivity in driving skin aging has not been systematically investigated. Previous studies have typically examined solar exposure and sensitive skin independently, without incorporating factorial experimental designs capable of evaluating interaction effects.

Therefore, the present study aimed to quantitatively evaluate the interactive effects of solar exposure and skin sensitivity on multiple skin aging parameters using a two‐factor factorial design combined with multimodal noninvasive skin measurement techniques. By integrating objective instrumental assessments with controlled clinical grouping, this study seeks to clarify whether individuals with sensitive skin are more susceptible to photoaging and to provide clinical evidence for targeted anti‐aging intervention strategies.

## 2 Methods

### 2.1 Study Participants and Grouping

This single‐center observational study enrolled 124 healthy Chinese female volunteers aged 25–34 years. All participants had been long‐term residents (≥10 years) of Luoyang, China, in order to minimize potential confounding effects of geographical and climatic variation on skin characteristics.

The sensitive‑skin group was required to meet all four of the following criteria:

1. Total stinging score ≥5 at 2.5 and 5 min after the lactic acid stinging test (LAST), performed using 10% lactic acid applied to the nasolabial fold [22].
2. Cheek TEWL [21] > 22 g/m^2^/h.
3. Moderate or higher sensitivity score on the Amorepacific proprietary sensitive skin self‐assessment questionnaire [23].
4. Self‐reported history of skin sensitivity lasting more than 5 years.

The non‑sensitive‑skin group was required to meet all four of the following criteria:

1. Negative lactic acid stinging test (LAST−).
2. Cheek TEWL [21] < 17 g/m^2^/h.
3. Nonsensitive score on the Amorepacific sensitive skin questionnaire [23].
4. No history of skin sensitivity.

Participants were also categorized according to occupational solar exposure:

Outdoor group: Approximately 4–5 h of occupational solar exposure per day.

Indoor group: Less than 2 h of occupational solar exposure per day.

Exclusion criteria included: severe systemic disease; use of corticosteroids, vasoconstrictors, antibiotics, or anti‑inflammatory agents within 2 weeks; history of severe allergy or cosmetic allergy in the past 1–2 years; pregnancy or lactation; participation in other trials within 2 months; prior cosmetic procedures on test sites; any other skin/medical condition affecting results; or judgement by investigators that the subject was unsuitable.

All participants provided written informed consent before enrollment. The study was conducted in accordance with the principles of the Declaration of Helsinki and Good Clinical Practice guidelines. The research protocol was approved by the Shanghai Clinical Research Ethics Committee (Approval No.: SECCR2025‐217‐01).

Based on skin sensitivity and solar exposure status, participants were divided into four groups (*n* = 31 per group): IS: mean age 30.35 ± 0.59 years; OS: mean age 29.65 ± 0.54 years; INS: mean age 30.35 ± 0.59 years; ONS: mean age 29.65 ± 0.54 years. No significant differences in mean age were observed among the four groups.

### 2.2 Noninvasive Skin Measurements

All measurements were conducted in a standardized climate‐controlled laboratory environment (temperature: 20–22°C; relative humidity: 40%–60%). Upon arrival, participants washed their faces using a standardized mild cleanser and rested in the testing room for at least 15 min to allow skin equilibration. During this period, participants completed the sensitive skin questionnaire.

Subsequently, trained technicians performed the LAST and TEWL measurements to confirm eligibility. After cleansing the nasal ala region, participants remained in the testing environment for an additional 30 min before baseline measurements were performed.

#### 2.2.1 Assessment of Skin Color and Gloss

Facial images were obtained using the VISIA‐CR imaging system (Canfield Scientific, Fairfield, USA). Image analysis was used to determine facial skin lightness (L* value), Individual Typology Angle (ITA°), and skin gloss parameters [24, 25, 26, 27]. All image processing was performed using Image‐Pro Plus 7.0 software.

#### 2.2.2 Assessment of Skin Wrinkles

Skin surface topography was measured using the Primos‐CR system (Canfield Scientific, USA), which employs digital stripe projection to quantify surface deformation. The resulting stripe distortions were analyzed using the system's proprietary software to derive quantitative wrinkle parameters [28].

#### 2.2.3 Assessment of Skin Surface Smoothness

Skin smoothness of the cheek area (SEsm) was evaluated using the Visioscan VC20 system (Courage+Khazaka, Cologne, Germany). This device utilizes high‐resolution ultraviolet light imaging to capture the active skin surface and analyze parameters related to surface morphology, roughness, and texture.

#### 2.2.4 Assessment of TEWL

TEWL was measured at the cheek region using a Tewameter (Courage+Khazaka, Cologne, Germany). The average of three consecutive measurements was recorded, with the unit expressed as grams per square metre per hour (g/m^2^/h).

#### 2.2.5 Assessment of Skin Hydration

Cheek skin hydration was quantified using a Corneometer (Courage+Khazaka, Köln, Germany), with outcomes in arbitrary units (a.u.).

#### 2.2.6 Assessment of Skin Elasticity

Cheek skin mechanical properties were evaluated via a Cutometer (Courage+Khazaka, Cologne, Germany) for two elasticity parameters: R2 (global elasticity, recovery after maximal negative pressure deformation) and F4 (skin firmness, suction‐induced deformation for quantifying tissue resistance).

#### 2.2.7 Assessment of Dermal Structure

Dermal microstructure was evaluated using a two‐photon microscope (Supervision‐780; Ultra‐View, China). This system utilizes two‐photon excited fluorescence and second harmonic generation imaging to visualize dermal structures in vivo [29, 30].

SCT: average distance from the skin surface to the first appearance of stratum granulosum cells in the fluorescence channel.

SHG: reflects collagen fiber organization and density.

AF: reflects the density and structural integrity of elastic fibers.

#### 2.2.8 Measurement of Sebum Content

Forehead sebum content was quantified via a Sebumeter (Courage+Khazaka, Köln, Germany) (measured after a 2‐hour waiting period), with values in µg/cm^2^.

### 2.3 Statistical Analysis

Continuous variables are presented as mean ± standard deviation (SD). For each of the 13 skin parameters, the effects of Solar exposure (indoor vs. outdoor), skin sensitivity (nonsensitive vs. sensitive), and their interaction were evaluated using two‐way analysis of variance (two‐way ANOVA). Partial eta squared (partial η^2^) was calculated to estimate effect sizes for the main and interaction effects. When a significant interaction between solar exposure and skin sensitivity was detected, simple main effects were examined using Bonferroni‐adjusted pairwise comparisons to identify differences between predefined groups while controlling the family‐wise error rate within each outcome variable.

## 3 Results

### 3.1 Baseline Characteristics of Participants

Baseline characteristics of the study population are summarized in Table 1. No significant differences were observed among the four groups in terms of age or duration of residence in the study area (*p* > 0.05).

**TABLE 1:** Mean ± SD for each parameter.

	Parameter	IS	INS	OS	ONS
1	L^*^	64.72±3.41	67.40±3.12	61.79±3.08	63.72±4.95
2	ITA°	44.54±11.44	50.51±10.42	40.42±8.68	43.58±13.62
3	Glossiness	0.051±0.02	0.049±0.018	0.05±0.017	0.045±0.025
4	Roughness	50.42±7.90	48.14±10.48	62.19±10.81	50.75±8.98
5	AF(%)	25.56±4.60	26.40±4.30	20.37±3.90	25.51±4.00
6	SHG(%)	42.70±4.50	44.10±4.30	35.86±3.30	40.64±5.70
7	SCT	9.10±1.99	9.16±2.05	6.45±1.34	8.97±1.99
8	Nasolabial Fold Length	29.77±6.93	30.77±8.70	37.81±14.45	29.74±11.13
9	Sebum	128.55±59.88	138.94±69.38	173.90±65.50	124.61±75.75
10	TEWL	26.17±3.47	15.13±1.31	30.12±5.73	16.03±0.71
11	R2	52.63±5.60	54.88±5.27	49.42±4.84	51.35±5.94
12	F4	5.88±0.87	5.65±0.81	6.54±1.03	6.11±0.92
13	Hydration	44.43±15.39	52.60±13.64	43.57±10.83	51.81±10.68

As expected based on the study design, daily occupational solar exposure differed significantly between indoor and outdoor groups (*p* < 0.001), confirming the validity of the environmental grouping.

### 3.2 Two‐Way ANOVA Results

A total of 13 skin parameters were analyzed, representing multiple aspects of skin physiology, including color, texture, structure, barrier function, and hydration status (Table 1). All data are presented as Mean ± SD.

Significant main effects of both solar exposure and skin sensitivity were observed for L^*^, ITA°, skin roughness, AF, SHG, stratum corneum thickness, TEWL, and the elasticity parameters R2 and F4. This indicates that these parameters are independently regulated by both extrinsic solar exposure and intrinsic skin sensitivity, indicating that these parameters are independently regulated by external solar environment and intrinsic skin sensitivity. Hydration showed a significant main effect only for skin sensitivity, whereas skin glossiness was not significantly affected by either factor under the present experimental conditions.

In addition to the main effects, significant interactions between solar exposure and skin sensitivity were detected for seven parameters: skin roughness, AF, SHG, stratum corneum thickness, Nasolabial Fold Length, Sebum  and TEWL (Table 2). This indicates that the impact of chronic solar exposure on specific skin‐aging characteristics is modulated by the individual's skin sensitivity status.

**TABLE 2:** Two‐way ANOVA results.

	Two‐way ANOVA results (*F*‐value / *p*‐value / ηp^2^)		
Parameter	Main effect A	Main effect B	Interaction effect A × B
L^*^	24.508/<0.001/0.170	11.883/<0.001/0.090	0.316/0.575/0.003
ITA°	7.568/0.007/0.059	5.174/0.025/0.041	0.487/0.486/0.004
Glossiness	0.554/0.458/0.005	0.636/0.427/0.005	0.149/0.700/0.001
Roughness	17.326/<0.001/0.126	15.761/<0.001/0.116	7.053/0.009/0.056
AF	16.086/<0.001/0.118	15.577/<0.001/0.115	8.099/0.005/0.063
SHG	39.705/<0.001/0.249	14.192/<0.001/0.106	4.434/0.037/0.036
SCT	17.948/<0.001/0.130	14.833/<0.001/0.110	13.387/<0.001/0.100
Nasolabial fold length	3.329/0.071/0.027	3.391/0.068/0.027	5.583/0.020/0.044
Sebum	1.620/0.206/0.013	2.546/0.113/0.021	5.992/0.016/0.048
TEWL	15.481/<0.001/0.114	415.970/<0.001/0.776	6.120/0.015/0.049
R2	11.954/<0.001/0.091	4.582/0.034/0.037	0.026/0.871/0.000
F4	11.774/<0.001/0.089	3.970/<0.049/0.032	0.361/0.549/0.003
Hydration	0.132/0.717/0.001	12.787/<0.001/0.096	0.000/0.989/0.000

The degrees of freedom are all (1, 120). When the interaction effect is significant, a simple effect analysis is conducted. All significant main and interaction effects remained unchanged after Benjamini–Hochberg false discovery rate (FDR) correction across the 39 ANOVA tests (Supplementary Table S1), supporting the robustness of the observed statistical associations. Interaction plots illustrating the significant interaction effects are presented in Figure 1.

**FIGURE 1:**
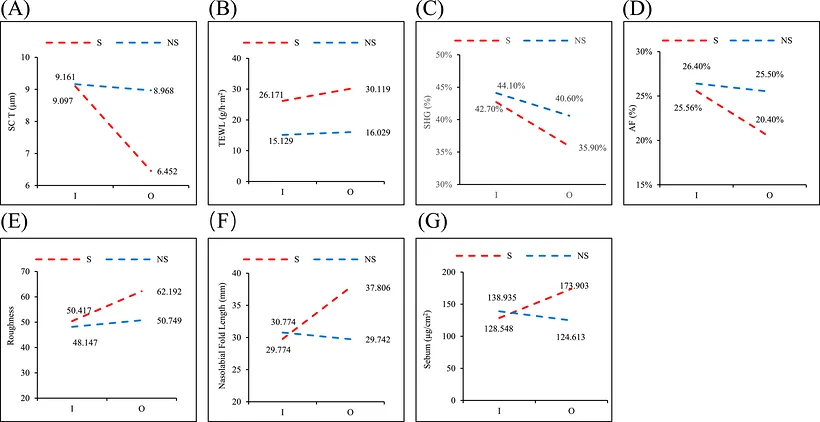
Interaction plots for skin parameters: stratum corneum thickness (A), TEWL (B), SHG (C), AF (D), Roughness (E), Nasolabial fold length (F), Sebum (G).

To further characterize these interaction effects, Bonferroni‐adjusted simple main effects analyses were subsequently performed. The detailed pairwise comparisons among the four experimental groups are summarized in Table 3. Overall, the outdoor‐sensitive (OS) group accounted for the majority of significant between‐group differences, suggesting that individuals with sensitive skin may exhibit greater susceptibility to the adverse effects of chronic solar exposure.

**TABLE 3:** Simple main effect pairwise comparisons with Bonferroni correction for skin parameters exhibiting significant solar exposure × skin sensitivity interaction effects.

Parameter	Comparison	Unadjusted *p‐*value	Bonferroni‐adjusted *p‐*value
AF	INS vs. IS	0.464	1.000
	INS vs. ONS	0.408	1.000
	INS vs. OS	<0.001	<0.001
	IS vs. ONS	0.965	1.000
	IS vs. OS	<0.001	<0.001
	ONS vs. OS	<0.001	<0.001
SHG	INS vs. IS	0.234	1.000
	INS vs. ONS	0.010	0.060
	INS vs. OS	<0.001	<0.001
	IS vs. ONS	0.119	0.715
	IS vs. OS	<0.001	<0.001
	ONS vs. OS	<0.001	<0.001
Roughness	INS vs. IS	0.340	1.000
	INS vs. ONS	0.298	1.000
	INS vs. OS	<0.001	<0.001
	IS vs. ONS	0.878	1.000
	IS vs. OS	<0.001	<0.001
	ONS vs. OS	<0.001	<0.001
Nasolabial Fold Length	INS vs. IS	0.618	1.000
	INS vs. ONS	0.686	1.000
	INS vs. OS	0.024	0.146
	IS vs. ONS	0.989	1.000
	IS vs. OS	0.008	0.047
	ONS vs. OS	0.017	0.101
Sebum	INS vs. IS	0.530	1.000
	INS vs. ONS	0.441	1.000
	INS vs. OS	0.046	0.274
	IS vs. ONS	0.821	1.000
	IS vs. OS	0.006	0.036
	ONS vs. OS	0.008	0.049
TEWL	INS vs. IS	<0.001	< 0.001
	INS vs. ONS	0.002	0.009
	INS vs. OS	<0.001	< 0.001
	IS vs. ONS	<0.001	< 0.001
	IS vs. OS	0.002	0.011
	ONS vs. OS	<0.001	< 0.001
SCT	INS vs. IS	0.900	1.000
	INS vs. ONS	0.706	1.000
	INS vs. OS	<0.001	< 0.001
	IS vs. ONS	0.799	1.000
	IS vs. OS	<0.001	< 0.001
	ONS vs. OS	<0.001	< 0.001

### 3.3 Skin Barrier Function

Skin barrier function was evaluated using stratum corneum thickness and TEWL.

Stratum corneum thickness showed the strongest interaction effect among all parameters (*F* = 13.397, *p* < 0.001, ηp^2^ = 0.100). Under outdoor conditions, stratum corneum thickness in sensitive skin (6.45 ± 1.34 µm) was significantly lower than that observed in nonsensitive skin (8.97 ± 1.99 µm). In contrast, no significant difference was observed between the IS group (9.10 ± 1.99 µm) and the INS group (9.16 ± 2.05 µm).

TEWL also demonstrated a significant interaction effect (*F* = 6.120, *p* = 0.015, ηp^2^ = 0.049). Sensitive skin exhibited higher TEWL values than nonsensitive skin under indoor conditions (26.17 ± 3.47 vs. 15.13 ± 1.31 g/m^2^/h). TEWL further increased in the OS group (30.12 ± 5.73 g/m^2^/h), representing the highest value among the four groups.

### 3.4 Dermal Matrix Integrity

The integrity of the dermal extracellular matrix (ECM) was evaluated using two key parameters acquired via two‐photon microscopy: second harmonic generation (SHG) signals, which specifically reflect the distribution and organisation of collagen fibers, and autofluorescence (AF) signals, which indicate the density and structural state of elastic fibers [31, 32]. SHG imaging offers distinct advantages over conventional histological techniques, as it enables high‐contrast visualization of collagen fibers without exogenous staining, thereby preserving tissue integrity and minimizing artefacts introduced by specimen preparation [33].

Representative two‐photon SHG images of the four participant groups are shown in Figure 2. In Figure 2, the red signal represents collagen fibers. The INS group displayed densely packed, well‐organized collagen fibers throughout the dermis, with a characteristic interwoven network structure consistent with the normal dermal ECM architecture of young, healthy skin [34]. The IS group exhibited a mild reduction in SHG signal intensity, with collagen fibers appearing slightly more fragmented and less densely arranged than in the INS group, suggesting that intrinsic barrier impairment in sensitive skin may be associated with subtle collagen alterations even in the absence of chronic solar exposure [21].

**FIGURE 2:**
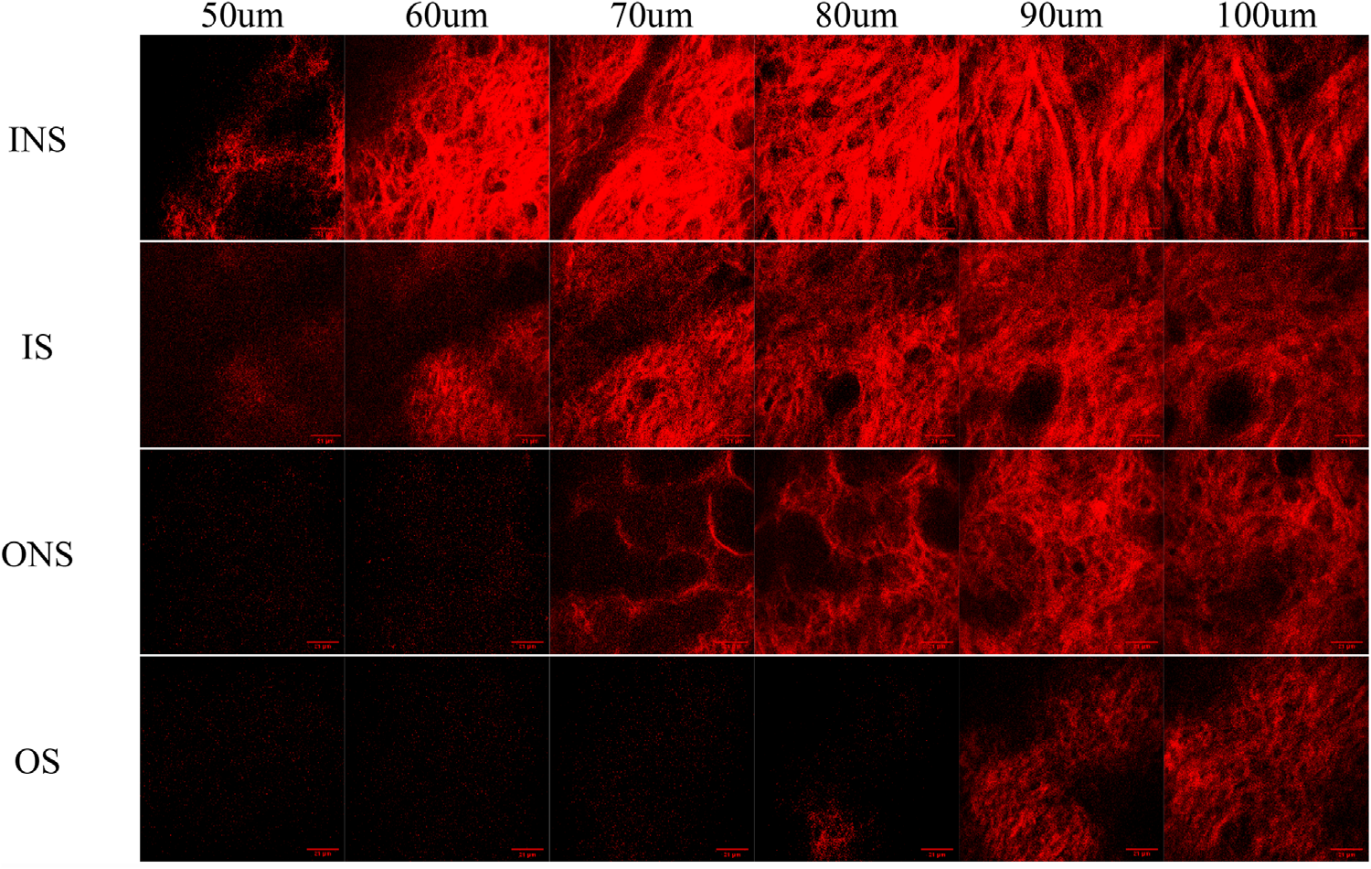
Representative second harmonic generation (SHG) images of dermal collagen fibers at the same depth from four participants. SHG signals, visualized in red, reflect the organisation and density of collagen fibers.

Quantitative analysis further confirmed the above morphological observations. SHG values demonstrated a significant interaction effect between solar exposure and skin sensitivity (*F* = 4.434, *p* = 0.037, ηp^2^ = 0.036). The SHG signal was significantly lower in the OS group (35.86% ± 3.30%) compared with the IS group (42.70% ± 4.50%) and the ONS group (40.64% ± 5.70%). The reduction in SHG signal reflects collagen fragmentation and density loss, likely resulting from MMP‑mediated collagen degradation induced by solar exposure [35]. Notably, the OS group showed a significantly greater decrease in SHG signal compared with the ONS group, which may be attributable to the fact that pre‐existing barrier defects and chronic low‐grade inflammation in sensitive skin exacerbate solar‐induced collagen degradation. The observation that collagen fiber fragmentation and disorganization precede and accompany wrinkle formation is consistent with previous findings that UVB‐induced alterations in collagen fiber orientation serve as an early trigger for skin wrinkling [36].

Representative two‐photon AF images of the four groups are presented in Figure 3. In Figure 3, the green signal represents elastic fibers. The INS group showed uniformly distributed, morphologically intact elastic fibers with a regular dendritic branching pattern, matching the normal morphological features of healthy skin elastic fibers [33]. The IS group displayed no obvious abnormalities in elastic fiber morphology compared with the INS group, and AF values (25.56% ± 4.60%) were essentially comparable to those of the INS group (26.40% ± 4.30%), indicating that sensitive skin per se does not significantly affect elastic fiber integrity in the absence of chronic long‑term solar exposure.

**FIGURE 3:**
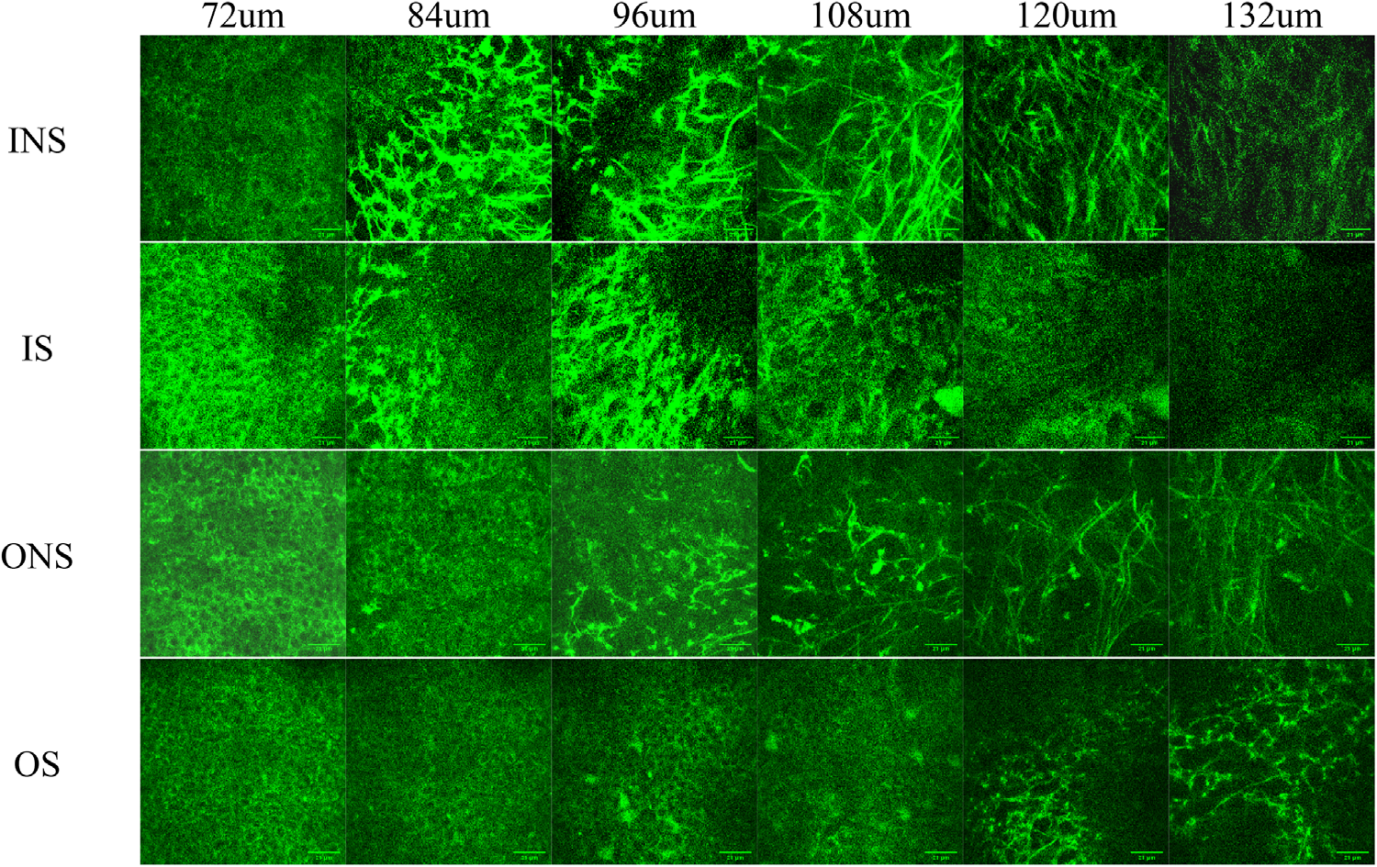
Representative autofluorescence (AF) images of dermal elastic fibres at the same depth from four participants. AF signals, visualized in green, reflect the density and structural integrity of elastic fibres.

The ONS group exhibited a mild decrease in elastic fiber signal, with some fibers showing a tendency towards thickening and aggregation; AF values (25.51% ± 4.00%) were slightly lower than those of the INS group, but the difference did not reach statistical significance, reflecting the progressive effects of early photoaging on elastic fibers [32].

In contrast, the OS group presented the most severe elastic fiber alterations, with a marked reduction in AF signal and residual elastic fibers displaying abnormal thickening, aggregation, and disorganization—characteristic features of severe elastin degradation and elastotic material accumulation, which are hallmarks of advanced photoaging [37].

Quantitative analysis further corroborated these findings. AF values revealed a significant interaction between solar exposure and skin sensitivity (*F* = 8.099, *p* = 0.005, ηp^2^ = 0.063). The OS group had the lowest AF value (20.37% ± 3.90%) among the four groups, significantly lower than that of the ONS group (25.51% ± 4.00%). Notably, AF values did not differ significantly between the ONS and INS groups, whereas the OS group showed significantly lower AF than both the IS and ONS groups, indicating that severe elastic fiber degradation is not driven by solar exposure or skin sensitivity alone, but rather by their synergistic interaction.  The significantly decreased AF signal in the OS group is indicative of severe elastic fiber disruption and degradation, a deterioration that is presumably attributable to the synergistic interplay between ultraviolet‑induced oxidative stress and the persistent inflammatory milieu inherent to sensitive skin [18].

### 3.5 Clinical Manifestations of Skin Aging

Clinical manifestations of skin aging were assessed using skin roughness and nasolabial fold length. Skin roughness demonstrated a significant interaction effect between solar exposure and skin sensitivity (*F* = 7.053, *p* = 0.009, ηp^2^ = 0.056). The OS group exhibited the highest roughness value (62.19 ± 10.81), which was significantly higher than that observed in the IS group (50.42 ± 7.90) and the ONS group (50.75 ± 8.98)

Similarly, nasolabial fold length showed a significant interaction effect between solar exposure and skin sensitivity (*F* = 5.583, *p* = 0.020, ηp^2^ = 0.044). The OS group presented the longest nasolabial fold length (37.81 ± 14.45 mm), which was significantly greater than that observed in the ONS group (29.74 ± 11.13 mm). No significant difference in nasolabial fold length was observed between the IS and INS groups.

### 3.6 Sebum Secretion

Sebum secretion was measured on the forehead using a Sebumeter. A significant interaction effect between solar exposure and skin sensitivity was observed for sebum secretion (*F* = 5.992, *p* = 0.016, ηp^2^ = 0.048).

The OS group exhibited the highest sebum level (173.90 ± 65.50 µg/cm^2^), which was significantly higher than that observed in the IS group (128.55 ± 59.88 µg/cm^2^).

In contrast, no significant difference in sebum secretion was observed between the INS group (138.94 ± 69.38 µg/cm^2^) and the ONS group (124.61 ± 75.75 µg/cm^2^).

## 4 Discussion

This study systematically investigated the interactive effects of solar exposure and skin sensitivity on multiple skin aging parameters using a two‐factor factorial design combined with multimodal noninvasive skin measurements. The results demonstrated significant interaction effects between solar exposure and skin sensitivity for several aging‐related parameters, including TEWL, stratum corneum thickness, SHG, AF, skin roughness, nasolabial fold length, and sebum secretion. Notably, individuals with sensitive skin exposed to outdoor solar environments exhibited the most pronounced aging‐related changes across multiple structural and functional parameters. These findings suggest that skin sensitivity may represent a phenotype with increased susceptibility to solar‐induced skin aging.

One of the most prominent findings of this study was the pronounced deterioration of epidermal barrier function observed in sensitive skin under solar exposure. The OS group exhibited both the lowest stratum corneum thickness and the highest TEWL among the four groups. Sensitive skin is widely recognized to exhibit intrinsic barrier impairment, often associated with altered lipid composition, reduced ceramide levels, and disrupted corneocyte organisation. When exposed to solar exposure, this pre‐existing barrier vulnerability may facilitate deeper penetration of environmental stressors and amplify epidermal damage [38]. Previous studies [39] have shown that solar exposure can induce keratinocyte DNA damage and apoptosis, while solar exposure promotes oxidative stress and disrupts lipid structures within the stratum corneum. The combined presence of intrinsic barrier fragility and chronic solar exposure may therefore create a reinforcing cycle of barrier disruption and environmental insult, ultimately contributing to accelerated skin aging.

In addition to epidermal barrier alterations, this study also revealed significant changes in dermal extracellular matrix integrity. Both SHG and AF signals, which reflect the structural organisation of collagen and elastic fibers respectively, were significantly reduced in the outdoor sensitive group. Multiphoton microscopy provides a powerful noninvasive approach for visualizing dermal structural changes associated with aging. Reduced SHG signals are generally interpreted as reflecting collagen fragmentation, reduced fiber density, or disorganization of collagen bundles. Similarly, decreased AF signals indicate degradation or structural disruption of elastic fibers within the dermal matrix. Chronic solar exposure is known to stimulate the production of reactive oxygen species (ROS), which activate matrix metalloproteinases (MMPs) responsible for collagen degradation. In individuals with sensitive skin, the presence of chronic low‐grade inflammation and impaired repair capacity may further exacerbate these processes, leading to more pronounced dermal matrix deterioration [35].

At the macroscopic level, these structural alterations were accompanied by more pronounced clinical manifestations of skin aging. The outdoor sensitive group exhibited both increased skin roughness and longer nasolabial folds compared with the other groups. Skin roughness reflects changes in surface microtopography, which are influenced by dermal structural support.

Meanwhile, nasolabial folds represent deeper wrinkle structures that are closely associated with dermal collagen degradation and reduced skin elasticity [40, 41]. The concurrent observation of decreased SHG/AF signals and increased wrinkle‐related parameters in the outdoor sensitive group suggests a consistent structure–phenotype relationship, in which dermal matrix deterioration may translate into visible clinical aging features.

Interestingly, this study also identified a significant interaction effect in sebum secretion. Sebum levels were markedly elevated in the OS group, whereas no comparable change was observed in nonsensitive skin under outdoor conditions. Although  the precise mechanisms underlying this phenomenon require further investigation, previous studies have suggested that solar exposure may influence sebaceous gland activity through inflammatory pathways or oxidative stress mechanisms. In sensitive skin, heightened neurovascular reactivity and inflammatory responses may contribute to an exaggerated sebaceous response under solar exposure.

Taken together, the present findings provide clinical evidence supporting the concept that sensitive skin may represent a phenotype with increased vulnerability to environmental aging factors. Rather than being solely a subjective sensory condition, sensitive skin appears to be associated with measurable structural and functional alterations that may predispose individuals to accelerated photoaging when exposed to chronic solar exposure.

This study has several strengths. First, the factorial study design allowed simultaneous evaluation of both independent and interaction effects of solar exposure and skin sensitivity. Second, the use of multimodal noninvasive measurement techniques enabled comprehensive assessment of skin aging across multiple biological levels, including epidermal barrier function, dermal matrix integrity, and clinical morphology. Third, the integration of advanced imaging technologies such as two‐photon microscopy provided in vivo visualization of dermal structural changes associated with photoaging.

Nevertheless, several limitations should also be acknowledged. First, this study employed a cross‐sectional design, which limits the ability to establish causal relationships between solar exposure, skin sensitivity, and aging outcomes. Second, solar exposure levels were estimated based on occupational exposure time rather than direct dosimetry measurements. Future studies incorporating wearable solar sensors may provide more precise exposure quantification. Third, the study population consisted exclusively of Chinese female participants within a relatively narrow age range, which may limit the generalizability of the findings to other populations.

Despite these limitations, the present study provides important insights into the interaction between skin sensitivity and solar exposure in the context of skin aging. The findings suggest that individuals with sensitive skin may require more targeted preventive strategies to mitigate photoaging risk. In addition to conventional photoprotection, interventions aimed at restoring epidermal barrier integrity and reducing inflammatory responses may be particularly beneficial for this population.

## 5 Conclusion

In conclusion, the present study demonstrates that the interaction between solar exposure and skin sensitivity plays a significant role in multiple aspects of skin aging. Sensitive individuals exposed to higher levels of solar exposure exhibited greater impairment in skin barrier function, reduced dermal collagen density, and more pronounced alterations in surface morphology and wrinkle characteristics.

These findings highlight the importance of considering both intrinsic skin sensitivity and environmental exposure when evaluating skin aging processes and developing personalized skincare and photoprotection strategies.

## Conflicts of Interest

The authors declare no conflicts of interest.

## Supporting information




**Supplementary Table S1**: FDR‐adjusted *p* values of two‐way ANOVA (39 tests, BenjaminiHochberg correction).

## Data Availability

The data that support the findings of this study are available from the corresponding author upon reasonable request.
