# Supplementary material for: Synergistic Effects of Solar Exposure and Skin Sensitivity on Skin Aging: A Clinical Study Using Multimodal Noninvasive Measurements
Source: Skin Res Technol. 2026 Oct 1;32(10):e70389. doi: 10.1111/srt.70389 (PMC13628154; doi:10.1111/srt.70389)
Supplement: Supplementary file 1 [file SRT-32-e70389-s001.docx]

| **Supplementary Table S1. FDR-adjusted P values of two-way ANOVA (39 tests, Benjamini–Hochberg correction)** | | | | | | |
| --- | --- | --- | --- | --- | --- | --- |
| Parameter | Effect | F | Raw_*P* | Partial_eta^2^ | FDR_*P* | Sig_change |
| L^*^ | Solar exposure | 24.508 | 0.000 | 0.170 | 0.000 | Unchanged |
|  | Skin sensitivity | 11.883 | 0.001 | 0.090 | 0.002 | Unchanged |
|  | Interaction | 0.316 | 0.575 | 0.003 | 0.641 | Unchanged |
| ITA^°^ | Solar exposure | 7.568 | 0.007 | 0.059 | 0.015 | Unchanged |
|  | Skin sensitivity | 5.174 | 0.025 | 0.041 | 0.042 | Unchanged |
|  | Interaction | 0.487 | 0.486 | 0.004 | 0.575 | Unchanged |
| Glossiness | Solar exposure | 0.554 | 0.458 | 0.005 | 0.558 | Unchanged |
|  | Skin sensitivity | 0.636 | 0.427 | 0.005 | 0.537 | Unchanged |
|  | Interaction | 0.149 | 0.700 | 0.001 | 0.758 | Unchanged |
| AF | Solar exposure | 16.086 | 0.000 | 0.118 | 0.001 | Unchanged |
|  | Skin sensitivity | 15.577 | 0.000 | 0.115 | 0.001 | Unchanged |
|  | Interaction | 8.099 | 0.005 | 0.063 | 0.012 | Unchanged |
| SHG | Solar exposure | 39.705 | 0.000 | 0.249 | 0.000 | Unchanged |
|  | Skin sensitivity | 14.192 | 0.000 | 0.106 | 0.001 | Unchanged |
|  | Interaction | 4.434 | 0.037 | 0.036 | 0.058 | Changed |
| Roughness | Solar exposure | 17.326 | 0.000 | 0.126 | 0.000 | Unchanged |
|  | Skin sensitivity | 15.761 | 0.000 | 0.116 | 0.001 | Unchanged |
|  | Interaction | 7.053 | 0.009 | 0.056 | 0.018 | Unchanged |
| SCT | Solar exposure | 17.948 | 0.000 | 0.130 | 0.000 | Unchanged |
|  | Skin sensitivity | 14.833 | 0.000 | 0.110 | 0.001 | Unchanged |
|  | Interaction | 13.387 | 0.000 | 0.100 | 0.001 | Unchanged |
| The length of the frown lines | Solar exposure | 3.329 | 0.071 | 0.027 | 0.098 | Unchanged |
|  | Skin sensitivity | 3.391 | 0.068 | 0.027 | 0.098 | Unchanged |
|  | Interaction | 5.583 | 0.020 | 0.044 | 0.035 | Unchanged |
|  |  |  |  |  |  |  |
| Sebum | Solar exposure | 1.620 | 0.206 | 0.013 | 0.267 | Unchanged |
|  | Skin sensitivity | 2.546 | 0.113 | 0.021 | 0.152 | Unchanged |
|  | Interaction | 5.992 | 0.016 | 0.048 | 0.029 | Unchanged |
| TEWL | Solar exposure | 15.481 | 0.000 | 0.114 | 0.001 | Unchanged |
|  | Skin sensitivity | 415.970 | 0.000 | 0.776 | 0.000 | Unchanged |
|  | Interaction | 6.120 | 0.015 | 0.049 | 0.029 | Unchanged |
| R2 | Solar exposure | 11.954 | 0.001 | 0.091 | 0.002 | Unchanged |
|  | Skin sensitivity | 4.582 | 0.034 | 0.037 | 0.056 | Changed |
|  | Interaction | 0.026 | 0.871 | 0.000 | 0.894 | Unchanged |
| F4 | Solar exposure | 11.774 | 0.001 | 0.089 | 0.002 | Unchanged |
|  | Skin sensitivity | 3.970 | 0.049 | 0.032 | 0.073 | Changed |
|  | Interaction | 0.361 | 0.549 | 0.003 | 0.630 | Unchanged |
| Hydration | Solar exposure | 0.130 | 0.719 | 0.001 | 0.758 | Unchanged |
|  | Skin sensitivity | 12.771 | 0.001 | 0.096 | 0.002 | Unchanged |
|  | Interaction | 0.000 | 0.990 | 0.000 | 0.990 | Unchanged |
